# Club cell-derived brain-derived neurotrophic factor regulates murine airway mechanics and mucin production in response to IL-13 in a sex-dependent manner

**DOI:** 10.3389/fphys.2025.1578553

**Published:** 2025-06-23

**Authors:** Amy Fagan, Mariana Sponchiado, Luz Mata, Shanil Amin, J. Ignacio Aguirre, Sreekala Prabhakaran, Leah R. Reznikov

**Affiliations:** ^1^ Department of Physiological Sciences, University of Florida, Gainesville, FL, United States; ^2^ Department of Pediatrics, Pediatric Pulmonary Division, University of Florida, Gainesville, FL, United States

**Keywords:** interleukin 13, club cell, mucin, brain-derived neurotrophic factor, airway mechanics

## Abstract

**Introduction:**

Brain-derived neurotrophic factor (BDNF) is a neural plasticity molecule that is increasingly recognized for its role in airway pathophysiology, including diseases like asthma. Although many cells in the airway can produce BDNF, our understanding of epithelial-derived BDNF and its role in airway health and disease remains limited.

**Methods:**

In the current study, we studied male and female mice with conditional loss of Bdnf in airway club cells and challenged them intranasally with saline (vehicle control) or interleukin 13 (IL-13) for 4 days. We measured pulmonary mechanics and the abundance and secretion characteristics of the major secreted mucin glycoproteins, mucin 5B (*Muc5b*) and mucin 5ac (*Muc5ac*).

**Results:**

Female mice with conditional loss of club cell *Bdnf* showed increased Muc5b protein in the airway epithelia under basal and IL-13-stimulated conditions compared to female mice with intact *Bdnf*. In contrast, conditional loss of club cell Bdnf in male mice augmented whole-lung *Muc5ac* mRNA levels under basal and IL-13-stimulated conditions. IL-13-treated female mice with conditional loss of club cell Bdnf showed decreased airway elastance in response to increasing concentrations of nebulized methacholine, suggesting that loss of club cell *Bdnf* had a protective effect. No statistically significant differences were observed in pulmonary mechanics between male mice with or without conditional loss of epithelial cell *Bdnf*, although treatment effects of IL-13 were noted. Mechanistic and complementary studies performed in NCI-H322 cells, a human cell line with “club cell-like” characteristics, failed to demonstrate a relationship among BDNF, IL-13 signaling, and *Muc5ac* at the mRNA level.

**Conclusion:**

These data highlight sex-dependent differences and club cell-specific effects of *Bdnf* in regulating airway physiology under inflammatory conditions in mice, suggesting that further studies are needed to understand potential translational implications.

## Introduction

Brain-derived neurotrophic factor (BDNF) is a prototypical neuroplasticity molecule originally identified for its ability to promote the survival of neurons (Barde et al., 1982). Over decades of research, it has become increasingly recognized that BDNF signaling is linked to pathologic changes in the respiratory system. For example, allergen provocation in asthmatic human subjects increases BDNF concentrations in the bronchoalveolar lavage fluid (Virchow et al., 1998). Polymorphisms in the *BDNF* gene are also associated with asthma (Szczepankiewicz et al., 2010; Szczepankiewicz et al., 2007; Wang et al., 2015; Andiappan et al., 2011).

Studies focused on BDNF in the context of asthma have emphasized its effects on airway smooth muscle and nerves. Notably, BDNF increases airway smooth muscle contractility (Prakash et al., 2006; Abcejo et al., 2012) and stimulates its proliferation (Liu et al., 2023). These findings suggest that BDNF may support asthma pathogenesis by promoting airway hyperresponsiveness (AHR). Studies have also found that BDNF is critical for innervation of the airway smooth muscle (Radzikinas et al., 2011; Aven and Ai, 2013), further suggesting a role for BDNF in supporting AHR. Sources of BDNF in the airway include airway smooth muscle (Ricci et al., 2004), neurons innervating the airway (Ricci et al., 2004; Zaidi et al., 2005), and airway epithelium (Hahn et al., 2006; Lommatzsch et al., 2003). Given that the airway epithelium is a critical interface to translate and coordinate inflammatory signals between the lung lumen and parenchyma, locally sourced BDNF may link airway insults with tissue responses.

BDNF cellular actions are mediated via two discrete receptors, namely, the high-affinity tropomyosin-related kinase B (TrkB, also known as NTRK2) receptor and the low-affinity receptor p75NTR (also known as NGFR) (De la Cruz-Morcillo et al., 2016). The TrkB receptor is the primary signaling receptor for BDNF, and binding of mature BDNF to the TrkB receptor leads to the activation of downstream pathways that regulate neuronal survival, differentiation, and plasticity (Wang et al., 2022). In contrast, the p75NTR receptor has a lower affinity for BDNF (De la Cruz-Morcillo et al., 2016) and is generally considered to be a co-receptor that enhances TrkB signaling in response to low concentrations of BDNF (Angoa-Perez et al., 2017).

Although our appreciation and understanding of BDNF in the airway are growing, gaps still remain to be addressed. One such gap is the role of epithelia-derived BDNF and subsequent impacts on airway health and disease. In the current study, we focused on club cells, a population of secretory cells in the small airways (Boers et al., 1999; Evans et al., 2004) that can repair the airway (Rawlins et al., 2009) and differentiate into mucus-secreting goblet cells (Chen et al., 2009) under inflammatory conditions. Excess production of mucin 5AC (Muc5ac) due to the expansion of goblet cells, alongside airway inflammation and airway remodeling, causes morbidity and mortality in asthma (Dunican et al., 2018). Given this, we tested the hypothesis that Bdnf derived from epithelia regulates airway inflammatory responses to interleukin 13 (IL-13), a key pro-inflammatory mediator of asthma pathogenesis (Wills-Karp, 2004).

## Materials and methods

### Animals

Both male and female mice heterozygous for floxed *Bdnf* (Rios et al., 2001) (strain 004339) were obtained from the Jackson Laboratory. These mice were bred to generate homozygous offspring. Homozygous offspring were then bred to mice purchased from the Jackson Laboratory [016225, B6N.129S6(Cg)-*Scgb1a1* ^tm1(cre/ERT)Blh^/J], which contain a tamoxifen-inducible Cre-recombinase under the control of secretoglobin family 1A member 1 (*Scgb1a1*) promoter. Scgb1a1, also known as Clara cell secretory protein (CCSP), expressed in airway club cells (Rawlins et al., 2009). Mice heterozygous or each gene were bred to produce mice homozygous for floxed *Bdnf* (Bdnf^fl/fl^) with (Scgb1a1^+^) or without (Scgb1a1^wt^) inducible *Cre*-recombinase. Breeding was carried out by the University of Florida Rodent Models Breeding Core. Adult male and female Bdnf^fl/fl^Scgb1a1^+^ and Bdnf^fl/fl^Scgb1a1^wt^ mice aged 8–10 weeks were used for the study. Mice were fed standard chow (2918, Teklad) and provided *ad libitum* access to water. They were housed on a 12-h light/dark cycle. Procedures were approved by and conducted in accordance with the University of Florida Institutional Animal Care and Use Committee.

### IL-13 and tamoxifen treatment

We used protocols previously established by our laboratory (Sponchiado et al., 2023; Sponchiado et al., 2024). In brief, Bdnf^fl/fl^Scgb1a1^+^ and Bdnf^l/fl^Scgb1a1^wt^ mice were anesthetized with 2% gaseous isoflurane using an induction chamber. Using a pipette, mice were then administered 50 µL of sterile IL-13 (50 μg/mL) in 0.9% saline or 50 µL of sterile 0.9% saline (vehicle control) intranasally for four consecutive days ^26, 27 28^. This paradigm of intranasal IL-13 results in significant goblet cell hypertrophy in the murine airway (Sponchiado et al., 2024; Pezzulo et al., 2019). All mice (Bdnf^fl/fl^Scgb1a1^+^ and Bdnf^fl/fl^Scgb1a1^wt^) received a 100-µL intraperitoneal injection of sterile tamoxifen in corn oil (20 mg/mL) on days 1 and 3. Tamoxifen induces *Cre*-recombinase activity and ablation of the floxed *Bdnf* fragment. This strategy was previously described by our laboratory (Sponchiado et al., 2023; Sponchiado et al., 2024).

### flexiVent

Approximately 20–24 h after the last IL-13 or vehicle control administration, mice were subjected to flexiVent procedures to investigate pulmonary mechanics as previously described (Sponchiado et al., 2023; Sponchiado et al., 2024; Reznikov et al., 2018; Schuller et al., 1991). In brief, mice were anesthetized using ketamine, xylazine, and acepromazine. Once a surgical plane of anesthesia was achieved, a tracheostomy was performed, and a blunted 18-g needle was inserted into the trachea. Ventilation parameters were set to 150 breaths/min with a volume of 10 mL/kg of body mass. A paralytic agent (rocuronium bromide) was administered, and baseline measurements were taken, followed by measurements in response to aerosolized methacholine (0–100 mg/mL). Aerosolization of methacholine was achieved using an ultrasonic nebulizer for a 10-s duration, as previously described (Sponchiado et al., 2023; Sponchiado et al., 2024; Reznikov et al., 2018; Schuller et al., 1991). At the end of the flexiVent experiment, mice were humanely euthanized under surgical plane anesthesia by cervical dislocation.

### Bronchoalveolar lavage and analyses

We performed lavage on the airway postmortem using three consecutive 1-mL lavages of sterile 0.9% saline. The material from the lavage was pooled and then spun down at 500 x g. We collected the supernatant and stored it at −80°C until further use. Granulocytes were determined using a hemocytometer and Kwik-Diff^TM^ Stain (99-907-00, Fisher Scientific).

### Enzyme-linked immunosorbent assay

We purchased Muc5ac (M7906) and Muc5b (M7978) enzyme-linked immunosorbent assays (ELISAs) from Biotang (Lexington, MA, United States). The bronchoalveolar lavages (BALs) were assayed in duplicate, and absorbance was measured using an accuSkan FC Microplate Photometer (Thermo Fisher Scientific, Waltham, MA, United States), as previously described (Sponchiado et al., 2023; Sponchiado et al., 2024). The specific details regarding the limits of sensitivity and intra-assay coefficients have been previously published by our laboratory (Sponchiado et al., 2023; Sponchiado et al., 2024).

### Histology

The left lung was removed and placed in 10% normal buffered formalin postmortem. None of the lungs were pressure-inflated or perfused with normal buffered formalin, and all lungs were collected following flexiVent procedures. The standardization of our sectioning procedure was previously published by our laboratory (Sponchiado et al., 2024) and was followed in the current study. We sectioned paraffin-embedded lungs at 4 μm thickness, as previously described (Sponchiado et al., 2023; Sponchiado et al., 2024). Sections with lower bronchioles were stained using a Periodic Schiff (PAS stain) (Epredia, cat. #87023). To visualize PAS-positive cells, we imaged the lungs using a Zeiss Axio Zoom.V16 microscope (Carl Zeiss, Germany). The number of PAS-positive cells was independently determined by two individuals blinded to treatment and genotype conditions. The number of PAS-positive cells was expressed as a density relative to airway epithelium. Measurements of the airway epithelia area were accomplished using ZenPro software (Carl Zeiss). The density of PAS-positive cells was averaged from the two independent observers. The mean density of PAS-positive cells per mouse was then used for statistical analysis.

### Immunohistochemistry

Procedures were carried out as previously published (Sponchiado et al., 2023; Sponchiado et al., 2024). In brief, 4 μm paraffin-embedded lung sections were deparaffinized and blocked for 30 min. Antigen retrieval was performed using heated 10 mM sodium citrate buffer (pH 6), as previously described. Immunolabeling for Muc5b (rabbit anti-Muc5b; HPA008246, Millipore Sigma; 1:500 dilution; 2 h) was performed using the Vectastain Elite ABC system (PK-6101, Vector Laboratories, CA, United States). The 3,3′-diaminobenzidine tetrachloride (34065, Thermo Fisher Scientific) method was used to visualize immunolabeling. Counterstaining with hematoxylin was performed. Cover-slipped slides were imaged using the Zeiss Axio Zoom.V16 microscope (Carl Zeiss). The mean intensity of Muc5b in the central airway was semi-quantified as previously described (Sponchiado et al., 2023; Sponchiado et al., 2024).

### NCI-H322 cell culture and treatment

We have previously described the human NCI-H322 cells (European Collection of Authenticated Cell Cultures; Sigma) and their culture conditions (Sponchiado et al., 2023; Sponchiado et al., 2024). These cells are a bronchoalveolar adenocarcinoma cell line with features characteristic of club cells (Schuller et al., 1991; Lau et al., 1987). After initial propagation, cells were assigned to treatments as described below.

#### NCI-H322 cells treated with the recombinant human BDNF protein

Cells were seeded onto 24-well plates. At 90% sub-confluency, cells were assigned to the following conditions: (i) 25 ng/mL recombinant human BDNF (n = 8) or (ii) saline vehicle control (n = 8). Recombinant BDNF or vehicle was diluted in complete media. The media were then replaced every 24 h with the abovementioned treatments for four consecutive days to mimic the duration of the *in vivo* study. After 4 days of treatment, cells were harvested with QIAzol (QIAGEN, Hilden, Germany), snap-frozen, and stored at −80°C until RNA isolation was performed. The dose of BDNF used is within the reported *in vitro* range across multiple cell types (Matsuda et al., 2012; Bartrup et al., 1997; Cheng et al., 2003). It is also within the range of reported circulating serum levels in humans (19–33 ng/mL) (Naegelin et al., 2018; Collins et al., 2021), although higher than the levels reported from sputum (–15 pg/mL) (Watanabe et al., 2015) or cultured human airway epithelial cells (–275 pg/mL) (Hahn et al., 2006).

#### NCI-H322 cells treated with recombinant human IL-13 and ANA-12

NCI-H322 cells were plated onto 24-well plates. At 90% sub-confluency, cells were assigned to the following conditions: (i) 20 ng/mL recombinant human IL-13 + 0.1% DMSO vehicle control (n = 8); (ii) 20 ng/mL recombinant human IL-13 + 100 nM ANA-12/0.1% DMSO (n = 8); (iii) saline vehicle control + 0.1% DMSO vehicle control (n = 8); or (iv) saline vehicle control + 100 nM ANA-12//0.1% DMSO (n = 8). ANA-12 is a TrkB antagonist (Cazorla et al., 2011). Drugs and chemicals were diluted in completed media. The medium was renewed every 24 h with the abovementioned treatments for four consecutive days. After 4 days of treatment, cells were harvested with QIAzol (QIAGEN, Hilden, Germany), snap-frozen, and stored at −80°C until RNA isolation. The concentration 20 ng/mL of IL-13 increases mucin in human airway cells *in vitro* (Thavagnanam et al., 2011; Zhen et al., 2007). The IC_50_ concentration of ANA-12 for the high-affinity TrkB receptor is 45.6 nM, whereas inhibition of the low-affinity p75NTR receptor occurs at 41.1 μM (Cazorla et al., 2011). Therefore, the dose used in our study is below the threshold for low-affinity receptor inhibition but within the range to completely abolish the activity of the high-affinity TrkB receptor.

### RNA isolation and qRT-PCR

The procedures for isolating RNA from the mouse lung and NCI-H322 cells have been previously described (Sponchiado et al., 2023; Sponchiado et al., 2024). In brief, the RNeasy Lipid Tissue Kit (QIAGEN) was used for isolation, and NanoDrop (Thermo Fisher Scientific) was used to assess RNA concentration and purity. A total of 2000 ng of total RNA was reverse-transcribed using Superscript VILO Master Mix (Thermo Fisher Scientific). Transcripts for *Muc5ac* and *Muc5b* were measured in murine lungs using primers based on *Mus musculus* mRNA GenBank (NCBI; www.ncbi.nlm.nih.gov), as previously published (Sponchiado et al., 2023; Sponchiado et al., 2024). Transcripts for *Muc5ac, BDNF, IL13RA1*, *IL4R*, and *IL13RA2* were quantified in NCI-H322 cells using primers based on *Homo sapiens* mRNA GenBank sequences. All primers are listed in Supplementary Table S1. Except for human *Muc5ac*, all have been previously published by our laboratory (Sponchiado et al., 2023; Sponchiado et al., 2024) (see Supplementary Table S1). PCR reactions were performed in triplicate using 96-well plates and Fast SYBR Green Master Mix (Applied Biosystems, Waltham, MA, United States). The cycling parameters used for PCR have been previously published by our laboratory and were followed in the current study (Sponchiado et al., 2023; Sponchiado et al., 2024). Primer pair products were visualized on an agarose gel as previously described (Sponchiado et al., 2023; Sponchiado et al., 2024). Relative abundances were calculated using the 2^−ΔΔCT^ method (Livak and Schmittgen, 2001). Actin beta (*Actb*) and ribosomal protein L13a (*RPL13A*) were used as endogenous controls for mouse and human PCR, respectively (Sponchiado et al., 2023; Sponchiado et al., 2024).

### End-point PCR for *Bdnf* mRNA

To perform end-point PCR, Platinum PCR SuperMix High Fidelity (Invitrogen) was used. The cycling parameters are as follows: initial denaturation at 94°C for 30 s, followed by 40 cycles of 15 s at 94°C, annealing at 50°C for 15 s, and extension at 68°C for 1 min and 30 s. The Bdnf^fl/fl^ mouse line has exon 5 of the *Bdnf* gene flanked by loxP sites, as previously described (Rios et al., 2001). Therefore, to assess recombination, primers that detected the wild-type and truncated *Bdnf* mRNA fragments were used (see Supplementary Table S1 for primers). PCR products were run on a 1% agarose gel, revealing both knockout and wild-type amplicons. Amplicons were gel-extracted and purified using the QIAquick Gel Extraction & Gel Cleanup Kit (QIAGEN). The purified DNA products were sequenced to confirm the recombination and excision of the floxed sequence. The endogenous wild-type gene amplicon is 1,400 bp (Supplementary Table S1). However, due to the insertion of loxP sites and cloning vector carryover (as shown in the original article), the amplified product before Cre-excision is 1,540 bp.

### RNAscope

The expression of *Bdnf* mRNA in lung tissue was assessed using the RNAscope Multiplex Fluorescent V2 Assay (catalog #323280, ACDBio), according to the manufacturer’s protocol. Dual-channel detection was achieved using a species-specific probe targeting *Bdnf* mRNA (catalog #457761-C2, ACDBio) and a probe specific for *Scgb1a1* mRNA, a marker of club cells (catalog #420351, ACDBio). Lung sections were cut on a cryostat at 12 μM and stored at −80°C until the day of the experiment. On the day of the experiment, sections were fixed in 4% paraformaldehyde (PFA) prepared in 1X PBS at 4°C for 15 min and subsequently dehydrated through a graded ethanol series (50%, 70%, and 100%). Pretreatment to enhance tissue permeability was performed by incubating sections with protease IV at room temperature for 30 min. Hybridization was carried out at 40°C for 2 h in the HybEZ Oven using the *Scgb1a1* probe (Channel 1) and the *Bdnf* probe (Channel 2). Signal amplification and detection were performed according to the manufacturer’s instructions, using TSA Vivid Fluorophore 520 to visualize green signals corresponding to Scgb1a1-positive club cells and TSA Vivid Fluorophore 570 to visualize red signals for *Bdnf* mRNA. Following amplification, sections were counterstained with DAPI at room temperature to visualize nuclei. Imaging and quantification were conducted as described below. A subset of lungs from seven (n = 4 female mice and n = 3 male mice) Bdnf ^fl/fl^Scgb1a1^wt^ mice and nine Bdnf ^fl/fl^Scgb1a1^+^ mice (n = 5 female mice and n = 4 male mice) were studied.

### Keyence imaging

Fluorescent imaging was performed using a Keyence BZ-X700 All-in-One Fluorescence Microscope equipped with a ×60 objective. Images were focused on small airway regions to capture *Bdnf* mRNA expression in *Scgb1a1*-positive club cells. To ensure consistency across samples, the exposure time for the red channel (TSA Vivid Fluorophore 570, *Bdnf* mRNA) was fixed at 5 s, while the green channel (TSA Vivid Fluorophore 520, *Scgb1a1*/club cell marker) and DAPI channel were adjusted to avoid signal saturation. Images were acquired using Keyence Analyzer software. Three to five airways were imaged per mouse for analysis.

### Semi-quantitative analysis of RNAscope

Keyence Images were imported into ImageJ. Using the ACD-recommended protocol for analysis, image thresholds were adjusted using Otsu’s method, and a binary image was generated. All mRNA molecules for *Bdnf* outside the *Scgb1a1*/club cell marker border were erased using the erase function, ensuring that only *Bdnf* mRNA particles arising from club cells were included in the analysis and not those from neighboring muscle, innervating nerves, or other cells. Image particle analysis was then conducted, and the integrated density was reported to quantify the probe signal intensity. The mean integrated density per mouse lung (representing 3–5 images per mouse lung) was used for statistical analysis.

### Chemicals and drugs

The use of acetyl-beta-methacholine-chloride (Sigma) for flexiVent studies (Sponchiado et al., 2023; Sponchiado et al., 2024) and mouse IL-13 (R&D Systems, MN, United States) (Sponchiado et al., 2024) has been previously described. Human IL-13 (R&D Systems, MN, United States) was reconstituted in sterile 0.9% saline with 0.1% BSA carrier to a 5 ng/mL concentration, aliquoted, and stored at −20°C until use. Fresh aliquots were brought to room temperature and used on the day of treatment. Recombinant human BDNF (R&D Systems, MN, United States) was reconstituted using sterile 0.9% saline with 0.1% BSA to a concentration of 100 μg/mL. It was also aliquoted and stored at −20°C until use. A fresh aliquot was used each day of treatment. ANA-12 (Selleckchem, cat. No. S7745) was diluted in DMSO to a concentration of 100 μΜ, aliquoted, and stored at −20°C until use. Fresh aliquots were used each day of treatment.

### Statistical analysis

A three-way ANOVA with sex, genotype, and treatment as the main factors, along with the double and triple interactions, was used to assess baseline flexiVent measurements. A three-way ANOVA was also performed for dynamic flexiVent measurements, using methacholine dose as a repeated measure, with genotype and treatment as main factors, and the resulting double and triple interactions were reported. Sexes were analyzed separately since there are well-documented sex differences in allergic asthma (Chowdhury et al., 2021). When the sexes were separated, a two-way ANOVA was used with genotype and treatment as the main factors. Significance was set at p < 0.05 for main effects and interactions. Sidak’s multiple comparisons test was used for post hoc analysis. For NCI-H322 cell studies, Student’s unpaired t-test was performed with significance set at p < 0.05. When two treatment groups were compared (BDNF vs. vehicle control), or when two factors were analyzed (IL-13 vs. vehicle and ANA-12 vs. vehicle), a two-way ANOVA was performed with significance set at p < 0.05. GraphPad Prism 10 was used for statistical analyses. Data are shown as the mean ± SEM.

## Results

### The *Bdnf* gene undergoes expected recombination, and transcripts are reduced in the airway

**FIGURE 1:**
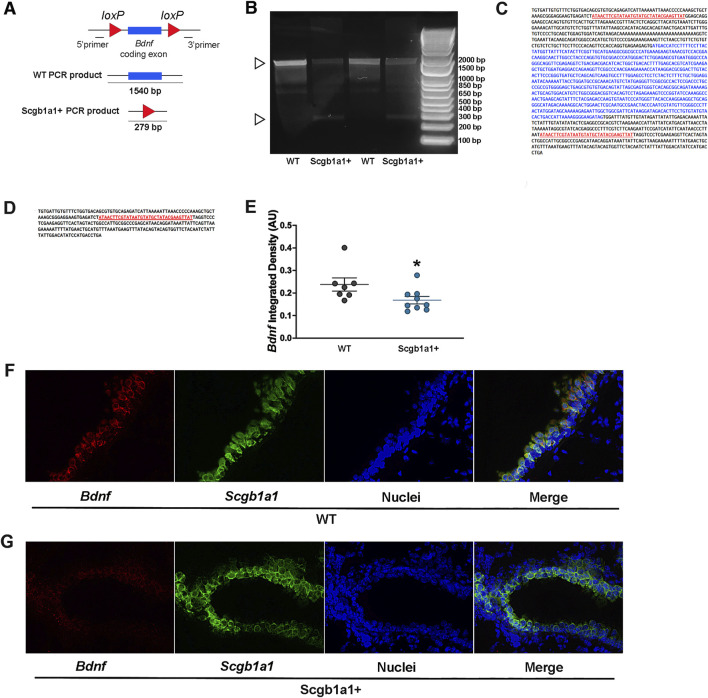
Cre-mediated recombination in the mouse lung. **(A)** *Bdnf* gene structure and recombination strategy. **(B)** cDNA from lung RNA samples was used as a template for PCR reactions to confirm the presence of truncated mRNA transcribed by Cre/lox-specific excision of *Bdnf* in Bdnf ^fl/fl^Scgb1a1^+^ mice. The 1,540 bp product represents the mRNA transcribed from the non-recombined *Bdnf*. A 279 bp fragment, which was detected in Bdnf^fl/fl^Scgb1a1^+^ samples, represents the truncated *Bdnf* mRNA when Cre excision occurred. The ladder concentration is greater than the concentration of the sample, thus resulting in the ladder to run faster. **(C)** Sequencing of the 1,540 bp fragment. **(D)** Sequencing of the 279 bp fragment, confirming lox-specific recombination. **(E)** Quantification of the *Bdnf* signal intensity in *Scgb1a1*-positive cells. Three to five images from wild-type lungs (n = 7 mice) and three to five images from Scgb1a1+ lungs (n = 9 mice) were examined. *, p < 0.05 compared to WT mice. **(F)** RNAscope of *Bdnf* (red) and *Scgb1a1* (green) in wild-type mouse lungs. Nuclei are shown in blue. **(G)** RNAscope of *Bdnf* (red) and *Scgb1a1* (green) in mouse lungs with conditional loss of club cell *Bdnf*. Nuclei are shown in blue. Abbreviations: WT, wild type; Scgb1a1^+^, club cell promoter-driving Cre recombinase; *Bdnf*, brain-derived neurotrophic factor.

The mice we studied have loxP sites flanking the *Bdnf* gene (Rios et al., 2001), resulting in a truncated *Bdnf* product upon *Cre*-lox recombination (Figure 1A). Previously, we reported that club cell recombination rates were approximately 75% (Sponchiado et al., 2023). To confirm recombination in the present study, mRNA expression of *Cre*-recombinase was first measured in Bdnf ^fl/fl^Scgb1a1^+^ mouse lungs using qRT-PCR. The mean Ct values for *Cre* mRNA were 20.74 ± 0.21 in male lungs (n = 12) and 20.37 ± 0.15 in female lungs (n = 11). The mean Ct value of *Cre* in Bdnf ^fl/fl^Scgb1a1^wt^ mice was considered absent (>35). These values were consistent with those in our prior work (Sponchiado et al., 2023; Sponchiado et al., 2024). Furthermore, recombination in Bdnf ^fl/fl^Scgb1a1^+^ mice was confirmed using end-point PCR in lung homogenates (Figure 1B). A smaller amplicon representing the recombined *Bdnf* gene was observed in Bdnf ^fl/fl^Scgb1a1^+^ lungs and not in Bdnf ^fl/fl^Scgb1a1^wt^ lungs (Figure 1B). A larger amplicon, representing the full-length gene, was found in both Bdnf ^fl/fl^Scgb1a1^wt^ and Bdnf^fl/fl^Scgb1a1^+^ lungs. Detection of the full-length amplicon in the Bdnf ^fl/fl^Scgb1a1^+^ lung homogenate was expected, given that (i) not all *Bdnf* in the lung homogenate originates from club cells and (ii) not all club cells undergo recombination. Sequencing confirmed the identity of both the small and large amplicons, as shown in Figures 1C,D. We also used antibodies to identify the Bdnf protein in the airways but were unable to confirm specific staining due to the presence of the signal in no primary control samples (data not shown). Therefore, RNAscope was performed to detect *Bdnf* and *Scgb1a1* mRNA in lung cross sections from Bdnf ^fl/fl^Scgb1a1^wt^ and Bdnf ^fl/fl^Scgb1a1^+^ mice. Semi-quantitative analysis (Figure 1D) showed that the intensity of the signal for *Bdnf* mRNA was reduced in Bdnf ^fl/fl^Scgb1a1^+^ mice (Figures 1E–G). Therefore, we concluded that recombination occurred, consistent with our previous reports.

### Conditional loss of club cell *Bdnf* dampens the effect of IL-13 on airway elastance in female mice

**FIGURE 2:**
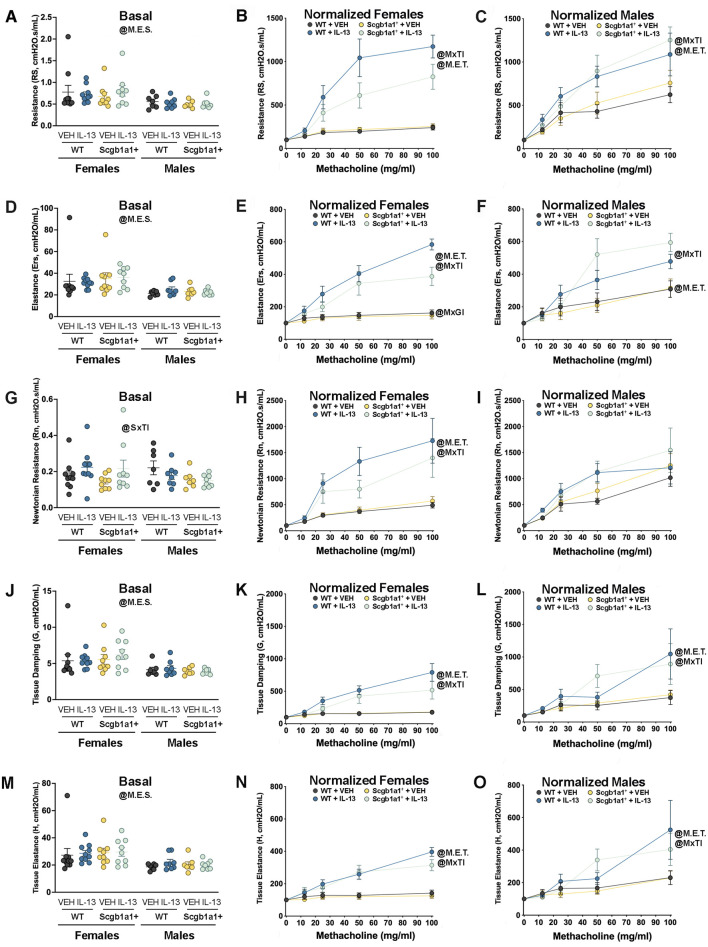
Conditional loss of club cell *Bdnf* regulates airway elastance in female mice. **(A)** Basal airway resistance in male and female mice. Normalized airway resistance in female **(B)** and male **(C)** airways. **(D)** Basal values of airway elastance in both male and female mice. Elastance normalized value in female **(E)** and male **(F)** mice. **(G)** Basal values of Newtonian resistance in male and female mice. Newtonian resistance normalized in female **(H)** and male **(I)** mice. **(J)** Baseline tissue damping in male and female mice. Tissue damping normalized in female **(K)** and male **(L)** mice. **(M)** Baseline tissue elastance values in male and female mice. Tissue elastance responses normalized in female **(N)** and male **(O)** mice. Female mice: Bdnf^fl/fl^Scgb1a1^wt^ mice treated with vehicle (n = 10) or IL-13 (n = 10); Bdnf^fl/fl^Scgb1a1^+^ mice treated with vehicle (n = 9) or IL-13 (n = 9). Male mice: Bdnf^ffl/fl^Scgb1a1^wt^ mice treated with vehicle (n = 7) or IL-13 (n = 8); Bdnf^ffl/fl^Scgb1a1^+^ mice treated with saline vehicle (n = 7) or IL-13 (n = 8). Abbreviations: WT, wild type; Scgb1a1^+^, club cell promoter-driving Cre recombinase; *Bdnf*, brain-derived neurotrophic factor; IL-13, interleukin 13; VEH, vehicle; @M.E.S., main effect of sex; @SxTI, sex × treatment interaction; @M.E.T., main effect of treatment; @MxTI, methacholine × treatment interaction; @MxGI, methacholine × genotype interaction. More information related to p values is available in Table 1.

A common feature of allergic asthma is AHR. Using single-frequency forced oscillation, we first examined total airway resistance. A main effect of sex (p = 0.0015, Figure 2A; Table 1) on basal airway resistance was observed, with male mice showing lower basal total airway resistance than female mice. Analysis of airway resistance in response to nebulized methacholine revealed a statistically significant methacholine × treatment interaction in female airways (p < 0.0001, Figure 2B; Table 1) and male airways (p = 0.0028, Figure 2C; Table 1). This statistically significant interaction reflected the expected increase in airway resistance in mice treated with IL-13. A non-significant statistical trend for genotype × treatment interaction was noted in female airways (p = 0.0719). Thus, in female airways, loss of club cell-derived *Bdnf* showed a non-significant trend toward reduced IL-13-induced AHR, while AHR in male airways remained unaffected.

**TABLE 1:** Summary of statistically significant main effects and interactions for airway mechanic studies.

Sex	Experimental outcome	Effect direction	Statistical parameter	p-value
Both	Basal airway resistance	↓ in male mice	Main effect of sex	0.0015
Male mice	Normalized airway resistance	↑ by IL-13	Main effect of treatment	0.0025
		↑ by IL-13	Methacholine × treatment interaction	0.0028
Female mice	Normalized airway resistance	↑ by IL-13	Main effect of treatment	<0.0001
		↑ by IL-13	Methacholine × treatment interaction	<0.0001
Both	Basal airway elastance	↓ in male mice	Main effect of sex	0.0002
Male mice	Normalized airway elastance	↑ by IL-13	Main effect of treatment	0.0050
		↑ by IL-13	Methacholine × treatment interaction	<0.0001
Female mice	Normalized airway elastance	↑ by IL-13	Main effect of treatment	<0.0001
		↑ by IL-13	Methacholine × treatment interaction	<0.0001
		↓ by genotype	Methacholine × genotype interaction	0.0474
Both	Basal Newtonian resistance	↑ by IL-13 in female mice but not in male mice	Sex × treatment interaction	0.0450
Male mice	Normalized Newtonian resistance	-	No effects of treatment or genotype	-
Female mice	Normalized Newtonian resistance	↑ by IL-13	Main effect of treatment	<0.0001
		↑ by IL-13	Methacholine × treatment interaction	<0.0001
Both	Basal tissue damping	↓ in male mice	Main effect of sex	0.0002
Male mice	Normalized tissue damping	↑ by IL-13	Main effect of treatment	0.0123
		↑ by IL-13	Methacholine × treatment interaction	0.0105
Female mice	Normalized tissue damping	↑ by IL-13	Main effect of treatment	<0.0001
		↑ by IL-13	Methacholine × treatment interaction	<0.0001
Both	Basal tissue elastance	↓ in male mice	Main effect of sex	0.0003
Male mice	Normalized tissue elastance	↑ by IL-13	Main effect of treatment	0.0235
		↑ by IL-13	Methacholine × treatment interaction	0.0057
Female mice	Normalized tissue elastance	↑ by IL-13	Main effect of treatment	<0.0001
		↑ by IL-13	Methacholine × treatment interaction	<0.0001

It was previously reported that IL-13 also increases airway elastance, which is the inverse of compliance (Sponchiado et al., 2024). Therefore, we measured airway elastance and found a main effect of sex (p = 0.0002, Figure 2D; Table 1), with female airways having higher basal airway elastance than male airways. When we examined normalized airway elastance following exposure to nebulized methacholine, we found a methacholine × genotype interaction (p = 0.0474, Figure 2E; Table 1), a methacholine × treatment interaction (p < 0.0001, Figure 2E; Table 1), and a trend for a methacholine × genotype × treatment interaction (p = 0.0790) in female airways. Notably, IL-13 increased airway elastance responses to methacholine, whereas the genotype decreased airway responses to methacholine. In male airways, a significant methacholine × treatment interaction (p < 0.0001, Figure 2F; Table 1) and a trend for a methacholine × genotype interaction (p = 0.0805) were noted. These statistical interactions were attributed to IL-13 increasing airway elastance in response to methacholine, with the genotype further amplifying this response. Therefore, loss of club cell *Bdnf* dampened the effect of IL-13 on dynamic airway elastance following methacholine exposure in female mouse airways only, with an opposite trend observed in male airways.

Using broadband frequency-forced oscillation, Newtonian resistance, tissue damping, and tissue elastance were also measured. A significant sex × treatment interaction was found in basal Newtonian resistance (p = 0.0450, Figure 2G; Table 1). The interaction suggested that sex modulated basal Newtonian resistance in response to IL-13, with female mice showing increases and male mice showing negligible changes in response to IL-13. Examination of normalized Newtonian resistance demonstrated a significant methacholine × treatment interaction in female airways (p < 0.0001, Figure 2H; Table 1) but not in male airways (Figure 2I). This statistical interaction was driven by IL-13-mediated increases in Newtonian resistance in response to methacholine in female airways.

Tissue damping is a measurement that is used to understand how energy is dissipated throughout the alveoli. Examination of basal tissue damping revealed a main effect of sex, with male airways having lower tissue damping than female mice (p = 0.0002, Figure 2J; Table 1). Assessment of dynamic tissue damping following exposure to methacholine demonstrated a significant methacholine × treatment interaction in both female airways (p < 0.0001, Figure 2K; Table 1) and male airways (p = 0.0105, Figure 2L; Table 1). These data suggested that IL-13 significantly elevated tissue damping following methacholine exposure in both sexes, but the loss of club cell *Bdnf* did not impact this.

Examination of basal tissue elastance revealed a main effect of sex (p = 0.0003, Figure 2M; Table 1), with male airways showing lower tissue elastance. A significant methacholine × treatment interaction in normalized tissue elastance in response to methacholine was also noted in both female mice (p < 0.0001, Figure 2N; Table 1) and male mice (p = 0.0057, Figure 2O; Table 1), with IL-13 treatment increasing tissue elastance responses. Thus, these data demonstrated that loss of club cell *Bdnf* had no impact on IL-13-mediated increases in tissue elastance under basal or methacholine-stimulated conditions.

### Loss of club cell *Bdnf* increases *Muc5ac* mRNA in male lungs and Muc5b protein levels in female lungs

**FIGURE 3:**
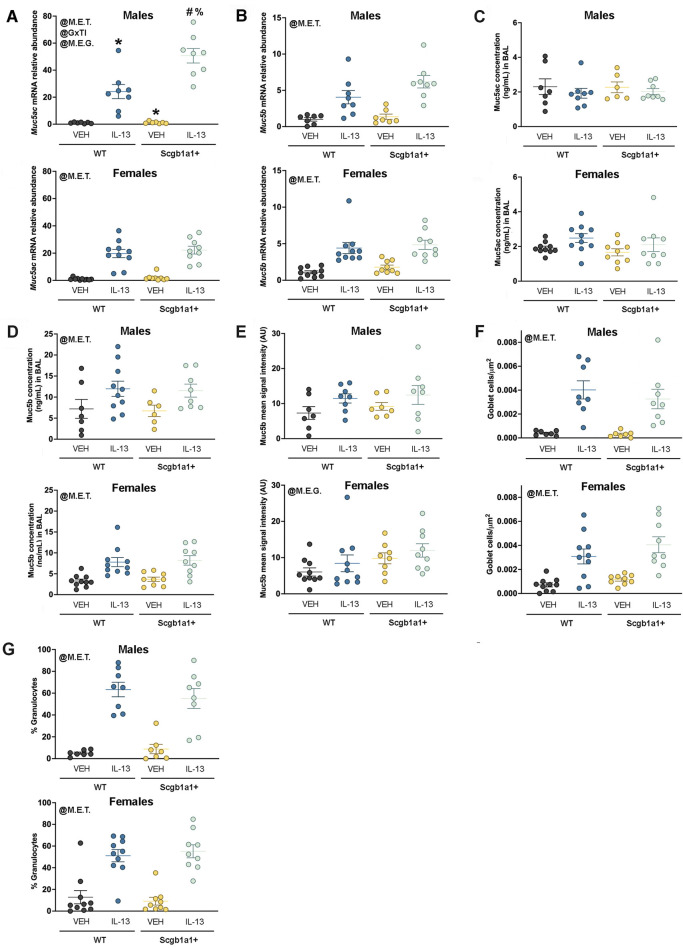
Loss of club cell Bdnf modifies mucin synthesis in a sex-dependent manner. **(A)** *Muc5ac* mRNA in male and female airways. For panel A, *, compared to WT + VEH; #, compared to WT + IL-13; and %, compared to Scgb1a1^+^ + VEH. **(B)** *Muc5b* mRNA in male and female airways. **(C)** Concentrations of Muc5ac in the BAL of male and female airways. **(D)** Concentration of Muc5b protein in the BAL of male and female airways. **(E)** Signal intensity of Muc5b immunolabeling in the airway surface in male and female airways. **(F)** Goblet cell density in male and female airways. **(G)** Percent of cells in the BAL that were granulocytes in male and female mice. Individual data points are collected from an individual mouse. Female mice: Bdnf^fl/fl^Scgb1a1^wt^ mice treated with vehicle (n = 10) or IL-13 (n = 10); Bdnf^fl/fl^Scgb1a1^+^ mice treated with vehicle (n = 9) or IL-13 (n = 9). Male mice: Bdnf^fl/fl^Scgb1a1^wt^ mice treated with vehicle (n = 7) or IL-13 (n = 8); Bdnf^fl/fl^Scgb1a1^+^ mice treated with vehicle (n = 7) or IL-13 (n = 8). Abbreviations: WT, wild type; Scgb1a1^+^, club cell promoter-driving Cre recombinase; *Bdnf*, brain-derived neurotrophic factor; IL-13, interleukin 13; VEH, vehicle; @M.E.T., main effect of treatment; @M.E.G., main effect of genotype; @GxTI, genotype × treatment interaction. More information regarding p values is available in Table 2.

A hallmark feature of IL-13-mediated inflammation in the airway is the increased production of mucins. Thus, we measured whole-lung mRNA expression of the major secreted mucin glycoproteins in the airway, *Muc5ac* and *Muc5b*. A significant treatment × genotype interaction (p = 0.0031, Figure 3A; Table 2) for *Muc5ac* mRNA expression was noted in male lungs. The interaction allowed for *post hoc* comparisons and demonstrated that IL-13 increased *Muc5ac* mRNA in Bdnf^fl/fl^Scgb1a1^wt^ and Bdnf^fl/fl^Scgb1a1^+^ mice but to a greater extent in mice with the loss of club cell *Bdnf.* The loss of club cell *Bdnf* also elevated basal *Muc5ac* mRNA in whole-lung male homogenates (Figure 3A). In female lungs, a main effect of treatment (p < 0.0001) was noted for *Muc5ac* mRNA, with the loss of club cell *Bdnf* having no impact on IL-13-stimulated or basal levels (Figure 3A; Table 2). For *Muc5b* mRNA, a treatment main effect was detected in male lungs (p < 0.0001, Figure 3B; Table 2), with a genotype trend noted (p = 0.0887). In female lungs, a main effect of treatment (p < 0.0001) on *Muc5b* mRNA was also observed (Figure 3B; Table 2). Thus, IL-13 increased transcription of *Muc5ac* and *Muc5b* mRNA independently of sex, but the loss of club cell *Bdnf* augmented the abundance of *Muc5ac* mRNA under basal and IL-13-stimulated conditions in male lungs only.

**TABLE 2:** Summary of statistically significant main effects and interactions for mucin studies.

Sex	Experimental outcome	Effect direction	Statistical parameter	p-value
Male mice	*Muc5ac* mRNA	↑ by genotype	Main effect of genotype	0.0028
		↑ by IL-13	Main effect of treatment	<0.0001
		↑ by genotype	Genotype × treatment interaction	0.0031
Female mice	*Muc5ac* mRNA	↑ by IL-13	Main effect of treatment	<0.0001
Male mice	*Muc5b* mRNA	↑ by IL-13	Main effect of treatment	<0.0001
Female mice	*Muc5b* mRNA	↑ by IL-13	Main effect of treatment	<0.0001
Male mice	Muc5ac protein in BAL	-	No effects of treatment or genotype	-
Female mice	Muc5ac protein in BAL	-	No effects of treatment or genotype	-
Male mice	Muc5b protein in BAL	↑ by IL-13	Main effect of treatment	0.0246
Female mice	Muc5b protein in BAL	↑ by IL-13	Main effect of treatment	<0.0001
Male mice	Muc5b immunohistochemistry signal intensity on airway surface	-	No effects of treatment or genotype	-
Female mice	Muc5b immunohistochemistry signal intensity on airway surface	↑ by genotype	Main effect of genotype	0.0246
Male mice	Goblet cells	↑ by IL-13	Main effect of treatment	<0.0001
Female mice	Goblet cells	↑ by IL-13	Main effect of treatment	<0.0001
Male mice	Granulocytes	↑ by IL-13	Main effect of treatment	<0.0001
Female mice	Granulocytes	↑ by IL-13	Main effect of treatment	<0.0001

To determine whether mucin protein levels also increased, we investigated Muc5ac and Muc5b protein concentrations in the BAL. There was no effect of treatment or genotype on BAL Muc5ac protein concentrations in male airways (Figure 3C; Table 2). In female airways, a trend was noted for IL-13 treatment to increase BAL Muc5ac protein concentrations (Figure 3C; Table 2). A main effect of IL-13 to increase Muc5b protein concentrations in the BAL of male mice (p < 0.0001, Figure 3D; Table 2) and female mice (p < 0.0001, Figure 3D; Table 2) was noted. Genotype did not affect BAL Muc5b protein concentrations in male or female mice.

Immunohistochemistry was performed on lung sections to further examine the potential impacts for loss of club cell *Bdnf* on mucin characteristics. Studies were performed on Muc5b due to its constitutive expression (Roy et al., 2014). A statistically non-significant trend toward increased Muc5b in male airways was observed with treatment (p = 0.0601, Figure 3E; Supplementary Figure S1A). Interestingly, a main effect of genotype for female airways (p = 0.0246, Figure 3E; Supplementary Figure S1B) was noted, with the loss of club cell *Bdnf* elevating levels of Muc5b. Finally, we interrogated the density of goblet cells using PAS staining techniques as a final method to examine the impact the loss of club cell *Bdnf* had on mucin characteristics. A main effect of IL-13 in increasing goblet cells was observed in both male airways (p < 0.0001, Figure 3F; Table 2; Supplementary Figure S2A) and female airways (p < 0.0001, Figure 3F; Table 2; Supplementary Figure S2B), with no effect of genotype. These results indicated that despite our findings that club cell *Bdnf* modified the abundance of *Muc5ac* mRNA in male airways and Muc5b protein in female airways, the IL-13-mediated elevation of goblet cell density was not impacted by the loss of club cell *Bdnf.* Thus, in female airways, the biophysical properties of Muc5b, such as retention and release, may be affected by the loss of club cell *Bdnf*. In male airways, however, the loss of club cell *Bdnf* may cause goblet cells to express more *Muc5ac* mRNA per cell.

Since the loss of club cell *Bdnf* regulated mucins in a gene-specific, sex-dependent, and treatment-dependent manner, we questioned whether the loss of club cell *Bdnf* also regulated the IL-13 inflammatory response from an immune cell perspective. The percentage of cells in the BAL that were granulocytes was increased by IL-13 in both male airways (p < 0.0001, Figure 3G; Table 2; Supplementary Figure S3A) and female airways (p < 0.0001, Figure 3G; Table 2; Supplementary Figure S3B), with no effect of genotype. Therefore, the loss of club cell *Bdnf* did not dampen the IL-13-mediated response of granulocytes entering the airway.

### BDNF does not regulate IL-13 signaling components or *Muc5ac* at the mRNA level in a human club cell-like line

**FIGURE 4:**
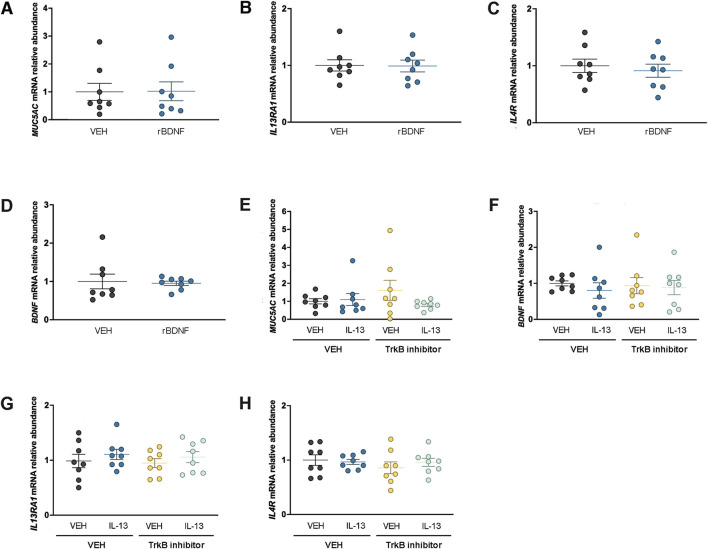
BDNF does not regulate *Muc5ac* or IL-13 receptors at the mRNA level in H322 cells. Expression of *Muc5ac* mRNA **(A)**, *IL13RA1* mRNA **(B)**, *IL4R* mRNA **(C)**, and *BDNF* mRNA **(D)** in H322 cells treated with vehicle or recombinant BDNF. Abundance of *Muc5ac* mRNA **(E)**, *BDNF* mRNA **(F)**, *IL13RA1* mRNA, **(G)** and *IL4R* mRNA **(H)** in H322 cells treated with recombinant IL-13 in the presence or absence of a TrkB inhibitor (ANA-12). For all panels, n = 8 per treatment group. Abbreviations: IL-13, interleukin 13; VEH, vehicle; BDNF, brain-derived neurotrophic factor; r, recombinant; TrkB, high-affinity tropomyosin-related kinase B

We previously reported that NCI-H322 cells, which have features of club cells (Schuller et al., 1991; Lau et al., 1987), provide a valuable complementary approach to our genetic mouse models (Sponchiado et al., 2023; Sponchiado et al., 2024). Our studies in male mouse airways suggested that the loss of club cell *Bdnf* augmented *Muc5ac* mRNA levels. Therefore, it was predicted that treating NCI-H322 cells with recombinant human BDNF for 4 days may reduce *Muc5ac* mRNA expression. However, no effect of BDNF was found on *Muc5ac* mRNA levels in NCI-H322 cells (Figure 4A). We also considered that BDNF could regulate IL-13 signaling components. Recombinant BDNF treatment did not alter *IL13RA1* mRNA expression (Figure 4B) or *IL4R* mRNA expression (Figure 4C). The mRNA for *IL13RA2* was below the threshold of detection (data not shown). Studies in other cell types have demonstrated that BDNF can regulate its own expression through positive feedback mechanisms (Esvald et al., 2020); therefore, we also investigated *BDNF* mRNA in NCI-H322 cells following treatment with recombinant BDNF. However, no effect of recombinant BDNF was found to regulate endogenous *BDNF* mRNA levels in NCI-H322 cells (Figure 4D).

To further examine the relationships among BDNF, IL-13, and mucin regulation, NCI-H322 cells were also treated with recombinant human IL-13 for 4 days in the presence or absence of an inhibitor to the high-affinity BDNF TrkB receptor. No significant effect of IL-13 or inhibition of the high-affinity TrkB receptor was found to regulate *Muc5ac* mRNA (Figure 4E). We focused on *Muc5ac* mRNA since the donor for NCI-H322 cells was male, and we found an effect of loss of club cell *Bdnf* on *Muc5ac* mRNA in male mouse airways. We considered that IL-13 may regulate BDNF expression but found no significant effect of recombinant IL-13 treatment or inhibition of the high-affinity TrkB receptor to regulate *BDNF* mRNA (Figure 4F). Finally, we considered whether either recombinant IL-13 or inhibition of TrkB could regulate IL-13 signaling components but found no effect of either treatment on *IL13RA1* (Figure 4G) *or IL4R* (Figure 4H) mRNA levels*.* The mRNA for *IL13RA2* was below the detection threshold (data not shown).

## Discussion

We tested the hypothesis that Bdnf derived from epithelia regulates airway inflammatory responses to IL-13. We generated a mouse model that allowed for conditional loss of club cell *Bdnf* and performed complementary studies in a human “club cell-like” NCI-H322 cell line. Our analysis of airway mechanics in mice revealed that reducing club cell *Bdnf* through conditional knockout had no major impacts on IL-13-mediated AHR, although a statistical trend for loss *Bdnf* to mitigate AHR was noted in female mice. It was observed, however, that conditional loss of club cell *Bdnf* blunted IL-13-mediated deficits in airway elastance in female mice but not in male mice. A reduction in elastance suggests that the airways were more distensible or more compliant. Therefore, one possibility is that the loss of club cell *Bdnf* reduced smooth muscle tone or contractility in response to IL-13. This speculation merits consideration given that the elimination of *Bdnf* from smooth muscle improves airway compliance in an experimental mouse model of allergic asthma (Britt et al., 2019). The three other airway mechanic properties we examined using broadband-forced oscillation revealed no impacts for the loss of club cell *Bdnf*, although sex- and treatment-specific effects were noted. The selective effects of the loss of club cell *Bdnf* on airway resistance and airway elastance versus tissue elastance and tissue damping align with the anatomic location of club cells and their reported functions in the airway (Dean and Snelgrove, 2018). It is also consistent with our prior report (Sponchiado et al., 2024).

Considering known sex differences in airway inflammatory responses and the baseline differences observed in our flexiVent data, we did not explicitly interrogate the interaction of sex, methacholine, genotype, and treatment. Nevertheless, it is noteworthy that vehicle-treated male mice qualitatively exhibited greater airway responses to methacholine than female mice. This observation aligns with findings by Card et al. (2006), who reported that male murine airways are more responsive to methacholine than their female counterparts.

Given that a hallmark feature of IL-13-mediated airway inflammation is increased production of mucins, several different assays were conducted to assess mucin production and expression at the molecular and cellular levels. It was found that the loss of club cell *Bdnf* increased *Muc5ac* mRNA abundance under basal and IL-13-stimulated conditions in male lungs only. This finding was consistent with our prior report demonstrating that the elimination of club cell Creb1 augmented *Muc5ac* mRNA (Sponchiado et al., 2024) in response to IL-13 in male mice only. Given that Creb1 regulates *Bdnf* (Esvald et al., 2020), it is possible that part of the mechanism explaining how the loss of club cell Creb1 regulates *Muc5ac* mRNA involves *Bdnf*. It is also possible that sex hormones, such as testosterone, intersect with Bdnf (Rasika et al., 1999) to regulate *Muc5ac* mRNA expression.

Although the loss of club *Bdnf* in male lungs augmented the abundance of *Muc5ac* mRNA, no parallel increase in Muc5ac protein concentrations in the BAL was observed. The disagreement between mRNA levels and protein concentrations may be due to alterations in mucin secretion mechanisms (Sponchiado et al., 2024). It is also possible that Muc5ac remains attached to the epithelium surface due to altered biophysical properties (Seibold, 2018). Similarly, no effect was found for loss of club cell *Bdnf* to modify the density of goblet cells. Thus, one speculation is that goblet cells in male mice with conditional loss of club cell *Bdnf* have a greater abundance of *Muc5ac* mRNA per goblet cell.

Interestingly, it was found that conditional elimination of club cell *Bdnf* enhanced the protein levels of Muc5b in female airways. The genotype-mediated increase in the Muc5b protein was not accompanied by parallel increases in BAL Muc5b protein concentrations or whole-lung *Muc5*b mRNA. Genotype-dependent increases were also not observed in goblet cell density. The reason for the selective effect of loss of club cell *Bdnf* in increasing Muc5b protein abundance in the airway remains unknown, but one speculation is that the loss of club cell *Bdnf* modifies secretion mechanisms or biophysical properties of Muc5b in a sex-dependent fashion. Estrogens are known to regulate BDNF expression in other tissues (Solum and Handa, 2002; Scharfman and MacLusky, 2006), as well as Muc5b in human airway cells (Choi et al., 2009). Therefore, estrogens may be a key intersection point to explain this observation. If additional studies confirm that club cell Bdnf decreases Muc5b protein levels in female airways, then it is possible that augmenting *Bdnf* expression or Bdnf signaling in female airways may have a beneficial effect in specific airway diseases.

To delineate potential mechanisms underlying BDNF regulation of mucins, we also performed studies in a human cell line with “club cell-like” characteristics. We performed two key pharmacologic experiments followed by molecular assessments: 1) treatment with recombinant BDNF and 2) treatment with recombinant IL-13 in the presence or absence of a high-affinity BDNF TrkB receptor inhibitor. We did not identify any role for BDNF in regulating *Muc5ac* mRNA in NCI-H322 cells or mRNA of IL-13 receptor components. The potential pathway activated by BDNF in club cells is uncertain. For example, BDNF displays context-specific signaling and can activate MAPK (Chen et al., 2017), PI3/Akt (Tecuatl et al., 2018), and PLC (Bathina and Das, 2015) pathways that also regulate *Muc5ac* in human airway cells (Wang et al., 2012; Chen et al., 2021; Damiano et al., 2018) *in vitro* or *in vivo*. Thus, given our *in vivo* findings in mice where the loss of club cell *Bdnf* regulated mucins and the crosstalk of BDNF signaling with mucin transcription, we anticipated that the treatment of NCI-H322 cells with recombinant BDNF would regulate *Muc5ac* mRNA. Since NCI-H322 cells are a non-small cell lung cancer line and TrkB signaling is intimately linked to tumor progression (Nakagawara et al., 1994; Sinkevicius et al., 2014), including non-small cell lung cancer (Chen et al., 2016), it is possible that BDNF signaling mechanisms are altered in NCI-H322 cells or that club cell-derived BDNF acts in a juxtacrine/paracrine fashion on other cell types that are not represented in the NCI-H322 monoculture model. If true, then such alterations may preclude us from understanding such mechanisms in human club cells. However, no other human “club cell-like” cell line is commercially available, we are currently limited to studying these cells. Potential altered regulation of BDNF–TrkB signaling may also explain the relatively little impact of TrkB inhibition observed in NCI-H322 cells under vehicle- or IL-13-stimulated conditions.

We also acknowledge that the dose of the TrkB inhibitor (ANA-12) used in our study was based on the IC_50_ established in neurons and recombinant cells overexpressing TrkB (Cazorla et al., 2011). In the same cells, the established ANA-12 IC50 for p75NTR was approximately 900 to 1900-fold greater. If NCI-H322 cells express p75NTR at levels 400–900-fold lower than neurons or recombinant cells, then it is possible that the ANA-12 dose we used (100 nM) also inhibited p75NTR. Yet, murine studies suggest that p75NTR expression is limited to nerves and immune cells throughout the lung (Nassenstein et al., 2007; Kerzel et al., 2003). Therefore, whether ANA-12 is interacting with p75NTR or whether p75NTR expression in NCI-H322 cells is at meaningful levels to trigger a significant cellular or molecular response remains to be determined. Alternatively, the data from the NCI-H322 cell line study may indicate that in human cells, there is no relationship among IL-13, BDNF, and mucins or that BDNF receptors are not expressed in these cells *in vitro*. Differences in the cellular actions of BDNF in human airways and Bdnf in mouse airways have also been reported by Britt et al. (2019). Another possibility is that the experimental conditions we implemented were not ideal for discerning such a relationship. Therefore, additional studies, such as those performed in differentiated airway epithelia, are needed to further evaluate the role of BDNF in regulating inflammatory responses in human airway epithelia/club cells.

Several limitations of our study warrant discussion. First, although we observed sex differences in mice, the underlying mechanisms responsible for these sex differences were not delineated. We also did not uncover the signaling mechanisms responsible for club cell-specific *Bdnf* regulation of airway mucins or airway mechanics. As highlighted, NCI-H322 cells are a non-small cell lung cancer cell line from a male donor, and BDNF–TrkB signaling is reported to be involved in the progression of non-small-cell lung cancer. Therefore, NCI-H322 cells may provide an incomplete or less-than-ideal model to explore mechanisms linking BDNF with the regulation of mucins and inflammation. These limitations highlight that future additional studies are needed.

In summary, our study is the first to identify club cell Bdnf as a molecule important for the regulation of airway mucin and airway mechanics. The sex-dependent effects we observed in mice provide further insights into the role of epithelial-derived Bdnf and its modulation of airway inflammatory responses. Such findings provide new knowledge to the airway biology field and may have implications for specific airway diseases where BDNF is suspected to play a role, including asthma.

## Data Availability

The original contributions presented in the study are included in the article/Supplementary Material, further inquiries can be directed to the corresponding author.

## References

[B1] Abcejo A. J. Sathish V. Smelter D. F. Aravamudan B. Thompson M. A. Hartman W. R. (2012). Brain-derived neurotrophic factor enhances calcium regulatory mechanisms in human airway smooth muscle. PLoS One 7 (8), e44343. 10.1371/journal.pone.0044343 22952960 PMC3430656

[B2] Andiappan A. K. Parate P. N. Anantharaman R. Suri B. K. Wang de Y. Chew F. T. (2011). Genetic variation in BDNF is associated with allergic asthma and allergic rhinitis in an ethnic Chinese population in Singapore. Cytokine 56 (2), 218–223. 10.1016/j.cyto.2011.05.008 21723144

[B3] Angoa-Perez M. Anneken J. H. Kuhn D. M. (2017). The role of brain-derived neurotrophic factor in the pathophysiology of psychiatric and neurological disorders. J. Psychiatry Psychiatr. Disord. 1 (5), 252–269. 10.26502/jppd.2572-519X0024 35098038 PMC8793768

[B4] Aven L. Ai X. (2013). Mechanisms of respiratory innervation during embryonic development. Organogenesis 9 (3), 194–198. 10.4161/org.24842 23974176 PMC3896590

[B5] Barde Y. A. Edgar D. Thoenen H. (1982). Purification of a new neurotrophic factor from mammalian brain. EMBO J. 1 (5), 549–553. 10.1002/j.1460-2075.1982.tb01207.x 7188352 PMC553086

[B6] Bartrup J. T. Moorman J. M. Newberry N. R. (1997). BDNF enhances neuronal growth and synaptic activity in hippocampal cell cultures. Neuroreport 8 (17), 3791–3794. 10.1097/00001756-199712010-00027 9427372

[B7] Bathina S. Das U. N. (2015). Brain-derived neurotrophic factor and its clinical implications. Arch. Med. Sci. 11 (6), 1164–1178. 10.5114/aoms.2015.56342 26788077 PMC4697050

[B8] Boers J. E. Ambergen A. W. Thunnissen F. B. (1999). Number and proliferation of clara cells in normal human airway epithelium. Am. J. Respir. Crit. Care Med. 159 (5 Pt 1), 1585–1591. 10.1164/ajrccm.159.5.9806044 10228131

[B9] Britt R. D. Jr. Thompson M. A. Wicher S. A. Manlove L. J. Roesler A. Fang Y. H. (2019). Smooth muscle brain-derived neurotrophic factor contributes to airway hyperreactivity in a mouse model of allergic asthma. FASEB J. 33 (2), 3024–3034. 10.1096/fj.201801002R 30351991 PMC6338659

[B10] Card J. W. Carey M. A. Bradbury J. A. DeGraff L. M. Morgan D. L. Moorman M. P. (2006). Gender differences in murine airway responsiveness and lipopolysaccharide-induced inflammation. J. Immunol. 177 (1), 621–630. 10.4049/jimmunol.177.1.621 16785560 PMC2262913

[B11] Cazorla M. Premont J. Mann A. Girard N. Kellendonk C. Rognan D. (2011). Identification of a low-molecular weight TrkB antagonist with anxiolytic and antidepressant activity in mice. J. Clin. Invest. 121 (5), 1846–1857. 10.1172/JCI43992 21505263 PMC3083767

[B12] Chen B. Liang Y. He Z. An Y. Zhao W. Wu J. (2016). Autocrine activity of BDNF induced by the STAT3 signaling pathway causes prolonged TrkB activation and promotes human non-small-cell lung cancer proliferation. Sci. Rep. 6, 30404. 10.1038/srep30404 27456333 PMC4960652

[B13] Chen G. Korfhagen T. R. Xu Y. Kitzmiller J. Wert S. E. Maeda Y. (2009). SPDEF is required for mouse pulmonary goblet cell differentiation and regulates a network of genes associated with mucus production. J. Clin. Invest. 119 (10), 2914–2924. 10.1172/JCI39731 19759516 PMC2752084

[B14] Chen T. Wu Y. Wang Y. Zhu J. Chu H. Kong L. (2017). Brain-derived neurotrophic factor increases synaptic protein levels via the MAPK/Erk signaling pathway and Nrf2/Trx Axis following the transplantation of neural stem cells in a rat model of traumatic brain injury. Neurochem. Res. 42 (11), 3073–3083. 10.1007/s11064-017-2340-7 28780733

[B15] Chen X. Yang J. Shen H. Zhang X. Wang H. Wu G. (2021). Muc5ac production inhibited by decreased lncRNA H19 via PI3K/Akt/NF-kB in asthma. J. Asthma Allergy 14, 1033–1043. 10.2147/JAA.S316250 34421304 PMC8373259

[B16] Cheng A. Wang S. Cai J. Rao M. S. Mattson M. P. (2003). Nitric oxide acts in a positive feedback loop with BDNF to regulate neural progenitor cell proliferation and differentiation in the mammalian brain. Dev. Biol. 258 (2), 319–333. 10.1016/s0012-1606(03)00120-9 12798291

[B17] Choi H. J. Chung Y. S. Kim H. J. Moon U. Y. Choi Y. H. Van Seuningen I. (2009). Signal pathway of 17beta-estradiol-induced MUC5B expression in human airway epithelial cells. Am. J. Respir. Cell Mol. Biol. 40 (2), 168–178. 10.1165/rcmb.2007-0377OC 18688042

[B18] Chowdhury N. U. Guntur V. P. Newcomb D. C. Wechsler M. E. (2021). Sex and gender in asthma. Eur. Respir. Rev. 30 (162), 210067. 10.1183/16000617.0067-2021 34789462 PMC8783601

[B19] Collins J. M. Hill E. Bindoff A. King A. E. Alty J. Summers M. J. (2021). Association between components of cognitive reserve and serum BDNF in healthy older adults. Front. Aging Neurosci. 13, 725914. 10.3389/fnagi.2021.725914 34408648 PMC8365170

[B20] Damiano S. Sasso A. De Felice B. Di Gregorio I. La Rosa G. Lupoli G. A. (2018). Quercetin increases MUC2 and MUC5AC gene expression and secretion in intestinal goblet cell-like LS174T via PLC/PKCα/ERK1-2 pathway. Front. Physiol. 9, 357. 10.3389/fphys.2018.00357 29681865 PMC5897515

[B21] Dean C. H. Snelgrove R. J. (2018). New rules for club development: new insights into human small airway epithelial club cell ontogeny and function. Am. J. Respir. Crit. Care Med. 198 (11), 1355–1356. 10.1164/rccm.201805-0925ED 29877729 PMC6290947

[B22] De la Cruz-Morcillo M. A. Berger J. Sanchez-Prieto R. Saada S. Naves T. Guillaudeau A. (2016). p75 neurotrophin receptor and pro-BDNF promote cell survival and migration in clear cell renal cell carcinoma. Oncotarget 7 (23), 34480–34497. 10.18632/oncotarget.8911 27120782 PMC5085170

[B23] Dunican E. M. Elicker B. M. Gierada D. S. Nagle S. K. Schiebler M. L. Newell J. D. (2018). Mucus plugs in patients with asthma linked to eosinophilia and airflow obstruction. J. Clin. Invest. 128 (3), 997–1009. 10.1172/JCI95693 29400693 PMC5824874

[B24] Esvald E. E. Tuvikene J. Sirp A. Patil S. Bramham C. R. Timmusk T. (2020). CREB family transcription factors are major mediators of BDNF transcriptional autoregulation in cortical neurons. J. Neurosci. 40 (7), 1405–1426. 10.1523/JNEUROSCI.0367-19.2019 31915257 PMC7044735

[B25] Evans C. M. Williams O. W. Tuvim M. J. Nigam R. Mixides G. P. Blackburn M. R. (2004). Mucin is produced by clara cells in the proximal airways of antigen-challenged mice. Am. J. Respir. Cell Mol. Biol. 31 (4), 382–394. 10.1165/rcmb.2004-0060OC 15191915 PMC10862391

[B26] Hahn C. Islamian A. P. Renz H. Nockher W. A. (2006). Airway epithelial cells produce neurotrophins and promote the survival of eosinophils during allergic airway inflammation. J. Allergy Clin. Immunol. 117 (4), 787–794. 10.1016/j.jaci.2005.12.1339 16630935

[B27] Kerzel S. Path G. Nockher W. A. Quarcoo D. Raap U. Groneberg D. A. (2003). Pan-neurotrophin receptor p75 contributes to neuronal hyperreactivity and airway inflammation in a murine model of experimental asthma. Am. J. Respir. Cell Mol. Biol. 28 (2), 170–178. 10.1165/rcmb.4811 12540484

[B28] Lau S. S. McMahon J. B. McMenamin M. G. Schuller H. M. Boyd M. R. (1987). Metabolism of arachidonic acid in human lung cancer cell lines. Cancer Res. 47 (14), 3757–3762. 3036346

[B29] Liu Q. Q. Tian C. J. Li N. Chen Z. C. Guo Y. L. Cheng D. J. (2023). Brain-derived neurotrophic factor promotes airway smooth muscle cell proliferation in asthma through regulation of transient receptor potential channel-mediated autophagy. Mol. Immunol. 158, 22–34. 10.1016/j.molimm.2023.04.004 37094390

[B30] Livak K. J. Schmittgen T. D. (2001). Analysis of relative gene expression data using real-time quantitative PCR and the 2(-Delta Delta C(T)) Method. methods 25 (4), 402–408. 10.1006/meth.2001.1262 11846609

[B31] Lommatzsch M. Braun A. Renz H. (2003). Neurotrophins in allergic airway dysfunction: what the mouse model is teaching us. Ann. N. Y. Acad. Sci. 992, 241–249. 10.1111/j.1749-6632.2003.tb03154.x 12794063

[B32] Matsuda S. Fujita T. Kajiya M. Takeda K. Shiba H. Kawaguchi H. (2012). Brain-derived neurotrophic factor induces migration of endothelial cells through a TrkB-ERK-integrin αVβ3-FAK cascade. J. Cell Physiol. 227 (5), 2123–2129. 10.1002/jcp.22942 21769870

[B33] Naegelin Y. Dingsdale H. Sauberli K. Schadelin S. Kappos L. Barde Y. A. (2018). Measuring and validating the levels of brain-derived neurotrophic factor in human serum. eNeuro 5 (2), ENEURO.0419–17.2018. 10.1523/ENEURO.0419-17.2018 29662942 PMC5898630

[B34] Nakagawara A. Azar C. G. Scavarda N. J. Brodeur G. M. (1994). Expression and function of TRK-B and BDNF in human neuroblastomas. Mol. Cell Biol. 14 (1), 759–767. 10.1128/mcb.14.1.759 8264643 PMC358424

[B35] Nassenstein C. Kammertoens T. Veres T. Z. Uckert W. Spies E. Fuchs B. (2007). Neuroimmune crosstalk in asthma: dual role of the neurotrophin receptor p75NTR. J. Allergy Clin. Immunol. 120 (5), 1089–1096. 10.1016/j.jaci.2007.07.007 17716721

[B36] Pezzulo A. A. Tudas R. A. Stewart C. G. Buonfiglio L. G. V. Lindsay B. D. Taft P. J. (2019). HSP90 inhibitor geldanamycin reverts IL-13- and IL-17-induced airway goblet cell metaplasia. J. Clin. Invest. 129 (2), 744–758. 10.1172/JCI123524 30640172 PMC6355221

[B37] Prakash Y. S. Iyanoye A. Ay B. Mantilla C. B. Pabelick C. M. (2006). Neurotrophin effects on intracellular Ca2+ and force in airway smooth muscle. Am. J. Physiol. Lung Cell Mol. Physiol. 291 (3), L447–L456. 10.1152/ajplung.00501.2005 16648236

[B38] Radzikinas K. Aven L. Jiang Z. Tran T. Paez-Cortez J. Boppidi K. (2011). A Shh/miR-206/BDNF cascade coordinates innervation and formation of airway smooth muscle. J. Neurosci. 31 (43), 15407–15415. 10.1523/JNEUROSCI.2745-11.2011 22031887 PMC3222097

[B39] Rasika S. Alvarez-Buylla A. Nottebohm F. (1999). BDNF mediates the effects of testosterone on the survival of new neurons in an adult brain. Neuron 22 (1), 53–62. 10.1016/s0896-6273(00)80678-9 10027289

[B40] Rawlins E. L. Okubo T. Xue Y. Brass D. M. Auten R. L. Hasegawa H. (2009). The role of Scgb1a1+ Clara cells in the long-term maintenance and repair of lung airway, but not alveolar, epithelium. Cell Stem Cell 4 (6), 525–534. 10.1016/j.stem.2009.04.002 19497281 PMC2730729

[B41] Reznikov L. R. Meyerholz D. K. Abou Alaiwa M. H. Kuan S. P. Liao Y. J. Bormann N. L. (2018). The vagal ganglia transcriptome identifies candidate therapeutics for airway hyperreactivity. Am. J. Physiol. Lung Cell Mol. Physiol. 315, L133–L148. 10.1152/ajplung.00557.2017 29631359 PMC6139658

[B42] Ricci A. Felici L. Mariotta S. Mannino F. Schmid G. Terzano C. (2004). Neurotrophin and neurotrophin receptor protein expression in the human lung. Am. J. Respir. Cell Mol. Biol. 30 (1), 12–19. 10.1165/rcmb.2002-0110OC 12791675

[B43] Rios M. Fan G. Fekete C. Kelly J. Bates B. Kuehn R. (2001). Conditional deletion of brain-derived neurotrophic factor in the postnatal brain leads to obesity and hyperactivity. Mol. Endocrinol. 15 (10), 1748–1757. 10.1210/mend.15.10.0706 11579207

[B44] Roy M. G. Livraghi-Butrico A. Fletcher A. A. McElwee M. M. Evans S. E. Boerner R. M. (2014). Muc5b is required for airway defence. Nature 505 (7483), 412–416. 10.1038/nature12807 24317696 PMC4001806

[B45] Scharfman H. E. MacLusky N. J. (2006). Estrogen and brain-derived neurotrophic factor (BDNF) in hippocampus: complexity of steroid hormone-growth factor interactions in the adult CNS. Front. Neuroendocrinol. 27 (4), 415–435. 10.1016/j.yfrne.2006.09.004 17055560 PMC1778460

[B46] Schuller H. M. Orloff M. Reznik G. K. (1991). Antiproliferative effects of the Ca2+/calmodulin antagonist B859-35 and the Ca(2+)-channel blocker verapamil on human lung cancer cell lines. Carcinogenesis 12 (12), 2301–2303. 10.1093/carcin/12.12.2301 1747931

[B47] Seibold M. A. (2018). Interleukin-13 stimulation reveals the cellular and functional plasticity of the airway epithelium. Ann. Am. Thorac. Soc. 15 (Suppl. 2), S98–S102. 10.1513/AnnalsATS.201711-868MG 29676620 PMC5955044

[B48] Sinkevicius K. W. Kriegel C. Bellaria K. J. Lee J. Lau A. N. Leeman K. T. (2014). Neurotrophin receptor TrkB promotes lung adenocarcinoma metastasis. Proc. Natl. Acad. Sci. U. S. A. 111 (28), 10299–10304. 10.1073/pnas.1404399111 24982195 PMC4104911

[B49] Solum D. T. Handa R. J. (2002). Estrogen regulates the development of brain-derived neurotrophic factor mRNA and protein in the rat hippocampus. J. Neurosci. 22 (7), 2650–2659. 10.1523/JNEUROSCI.22-07-02650.2002 11923430 PMC6758321

[B50] Sponchiado M. Bonilla A. L. Mata L. Jasso-Johnson K. Liao Y. J. Fagan A. (2023). Club cell CREB regulates the goblet cell transcriptional network and pro-mucin effects of IL-1B. Front. Physiol. 14, 1323865. 10.3389/fphys.2023.1323865 38173934 PMC10761479

[B51] Sponchiado M. Fagan A. Mata L. Bonilla A. L. Trevizan-Bau P. Prabhakaran S. (2024). Sex-dependent regulation of mucin gene transcription and airway secretion and mechanics following intra-airway IL-13 in mice with conditional loss of club cell Creb1. Front. Physiol. 15, 1392443. 10.3389/fphys.2024.1392443 38711951 PMC11070562

[B52] Szczepankiewicz A. Breborowicz A. Skibinska M. Wilkosc M. Tomaszewska M. Hauser J. (2007). Association analysis of brain-derived neurotrophic factor gene polymorphisms in asthmatic children. Pediatr. Allergy Immunol. 18 (4), 293–297. 10.1111/j.1399-3038.2007.00525.x 17584309

[B53] Szczepankiewicz A. Breborowicz A. Sobkowiak P. Popiel A. (2010). Association of BDNF gene polymorphism with asthma in polish children. World Allergy Organ J. 3 (9), 235–238. 10.1097/WOX.0b013e3181eedb68 23282800 PMC3651092

[B54] Tecuatl C. Herrrera-Lopez G. Martin-Avila A. Yin B. Weber S. Barrionuevo G. (2018). TrkB-mediated activation of the phosphatidylinositol-3-kinase/Akt cascade reduces the damage inflicted by oxygen-glucose deprivation in area CA3 of the rat hippocampus. Eur. J. Neurosci. 47 (9), 1096–1109. 10.1111/ejn.13880 29480936 PMC5938095

[B55] Thavagnanam S. Parker J. C. McBrien M. E. Skibinski G. Heaney L. G. Shields M. D. (2011). Effects of IL-13 on mucociliary differentiation of pediatric asthmatic bronchial epithelial cells. Pediatr. Res. 69 (2), 95–100. 10.1203/PDR.0b013e318204edb5 21076368

[B56] Virchow J. C. Julius P. Lommatzsch M. Luttmann W. Renz H. Braun A. (1998). Neurotrophins are increased in bronchoalveolar lavage fluid after segmental allergen provocation. Am. J. Respir. Crit. Care Med. 158 (6), 2002–2005. 10.1164/ajrccm.158.6.9803023 9847299

[B57] Wang C. S. Kavalali E. T. Monteggia L. M. (2022). BDNF signaling in context: from synaptic regulation to psychiatric disorders. Cell 185 (1), 62–76. 10.1016/j.cell.2021.12.003 34963057 PMC8741740

[B58] Wang G. Xu Z. Wang R. Al-Hijji M. Salit J. Strulovici-Barel Y. (2012). Genes associated with MUC5AC expression in small airway epithelium of human smokers and non-smokers. BMC Med. Genomics 5, 21. 10.1186/1755-8794-5-21 22676183 PMC3443416

[B59] Wang J. Y. Wang A. L. Han W. Mu Z. L. (2015). Association between a functional single nucleotide polymorphism in the brain-derived neurotrophic factor gene and risk of child asthma. Genet. Mol. Res. 14 (4), 16233–16240. 10.4238/2015.December.8.13 26662416

[B60] Watanabe T. Fajt M. L. Trudeau J. B. Voraphani N. Hu H. Zhou X. (2015). Brain-derived neurotrophic factor expression in asthma. Association with severity and type 2 inflammatory processes. Am. J. Respir. Cell Mol. Biol. 53 (6), 844–852. 10.1165/rcmb.2015-0015OC 25945802 PMC4742942

[B61] Wills-Karp M. (2004). Interleukin-13 in asthma pathogenesis. Immunol. Rev. 202, 175–190. 10.1111/j.0105-2896.2004.00215.x 15546393

[B62] Zaidi S. I. Jafri A. Doggett T. Haxhiu M. A. (2005). Airway-related vagal preganglionic neurons express brain-derived neurotrophic factor and TrkB receptors: implications for neuronal plasticity. Brain Res. 1044 (2), 133–143. 10.1016/j.brainres.2005.02.037 15885212

[B63] Zhen G. Park S. W. Nguyenvu L. T. Rodriguez M. W. Barbeau R. Paquet A. C. (2007). IL-13 and epidermal growth factor receptor have critical but distinct roles in epithelial cell mucin production. Am. J. Respir. Cell Mol. Biol. 36 (2), 244–253. 10.1165/rcmb.2006-0180OC 16980555 PMC1899314

