# Supplementary figures and images for: Club cell-derived brain-derived neurotrophic factor regulates murine airway mechanics and mucin production in response to IL-13 in a sex-dependent manner
Source: Front Physiol. 2025 Jun 23;16:1578553. doi: 10.3389/fphys.2025.1578553 (PMC12230438; doi:10.3389/fphys.2025.1578553)

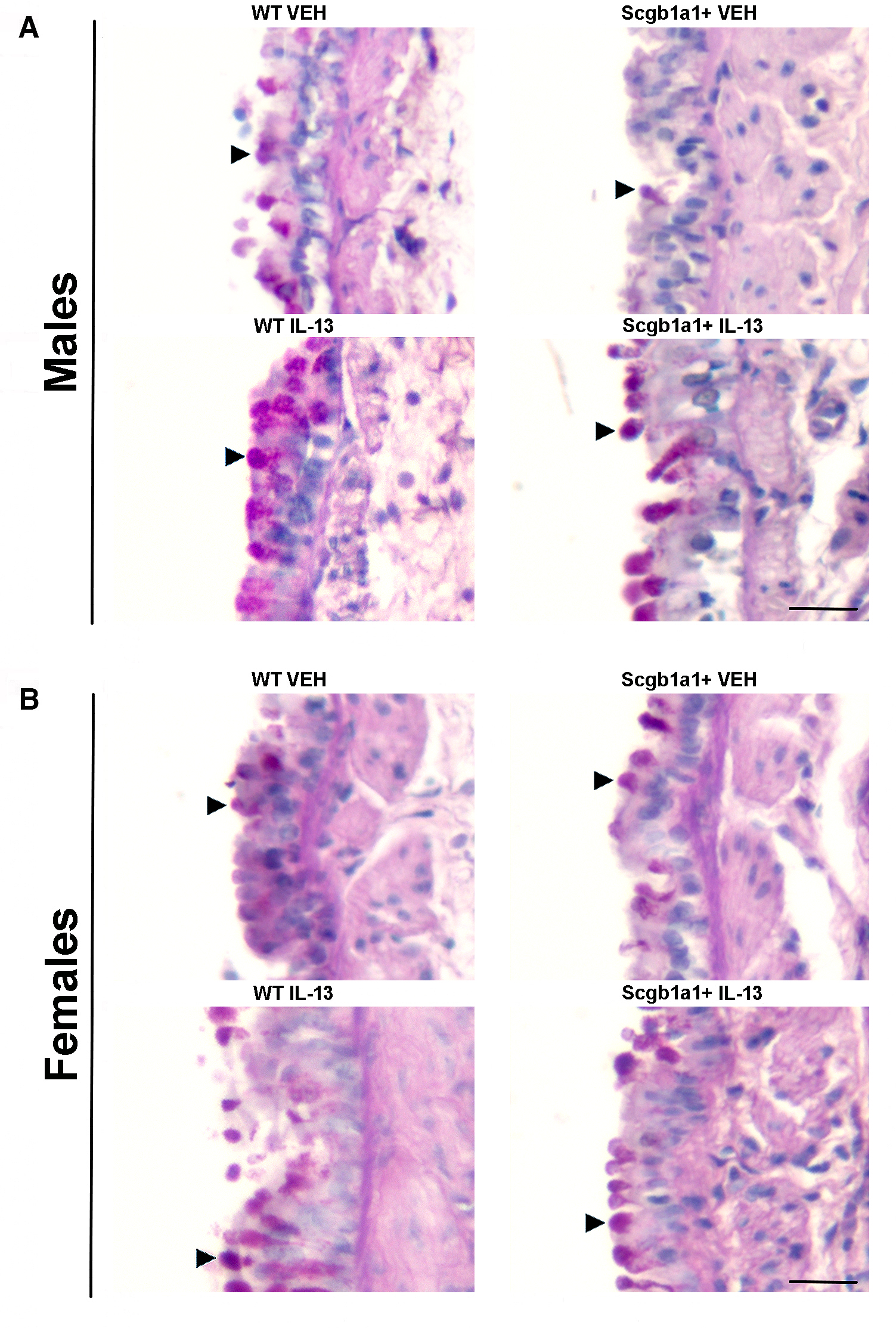

Supplement: Supplementary file 1 [file Supplementaryfile2.JPEG]

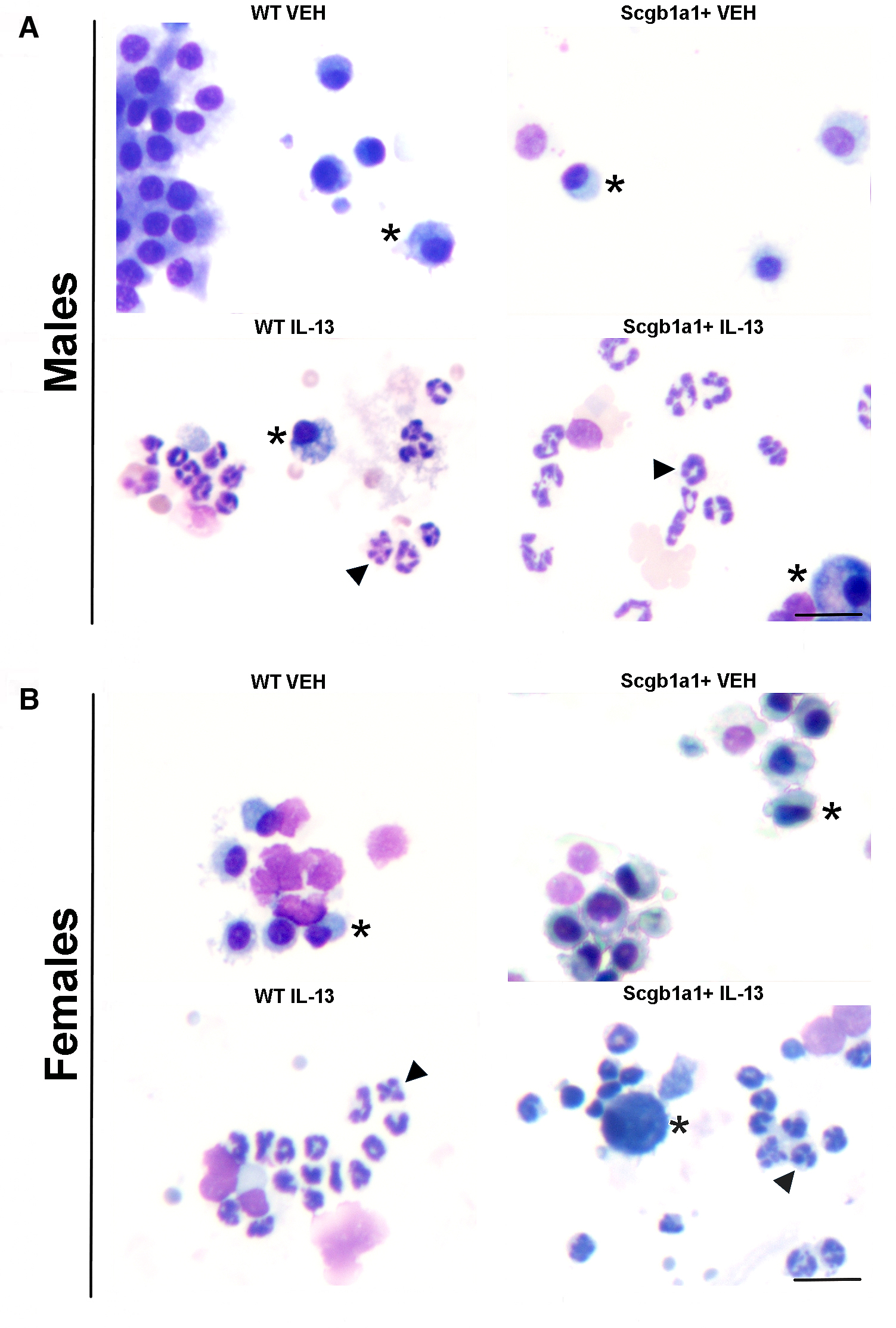

Supplement: Supplementary file 3 [file Supplementaryfile3.JPEG]

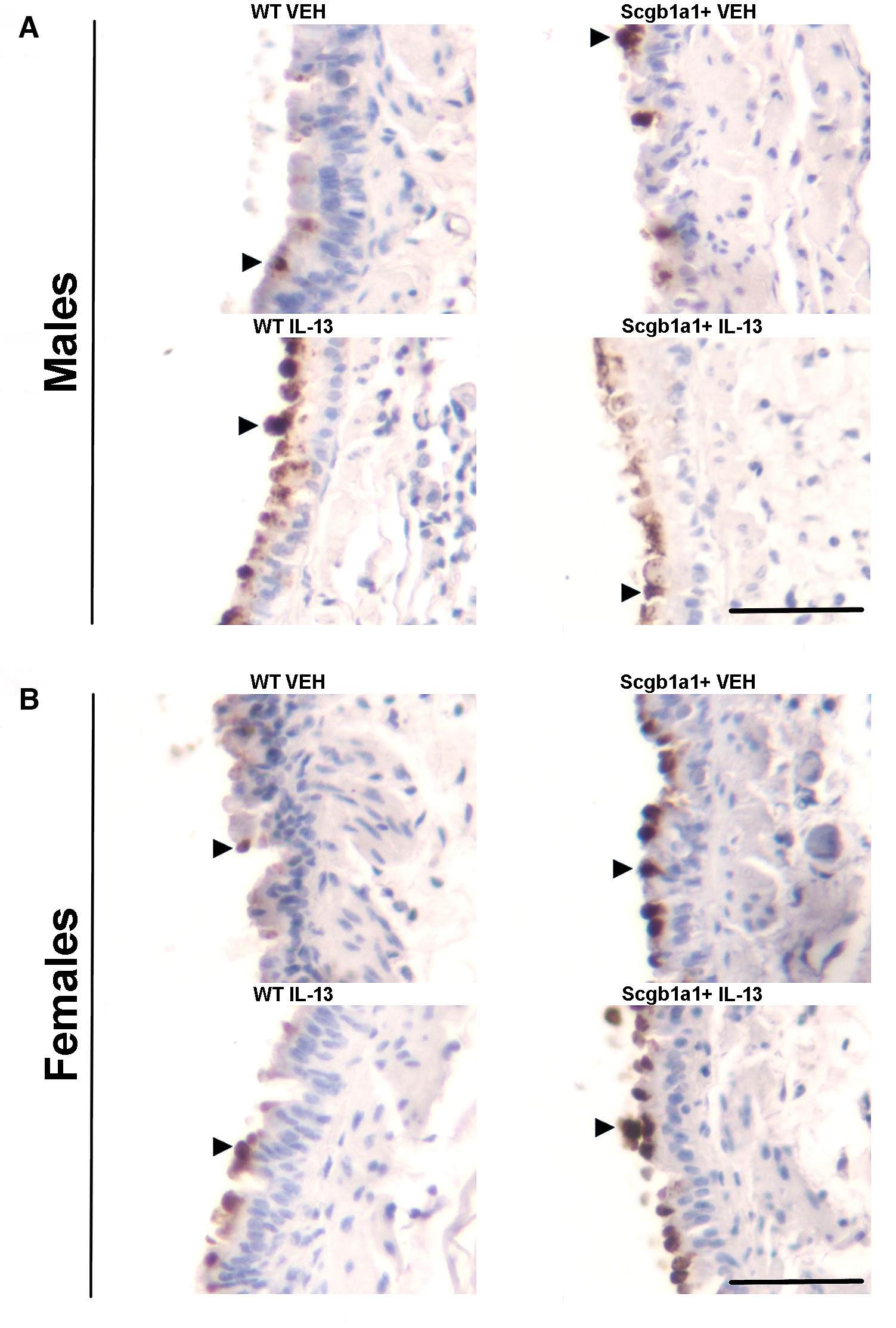

Supplement: Supplementary file 4 [file Supplementaryfile1.JPEG]
