# Supplementary material for: Club cell-derived brain-derived neurotrophic factor regulates murine airway mechanics and mucin production in response to IL-13 in a sex-dependent manner
Source: Front Physiol. 2025 Jun 23;16:1578553. doi: 10.3389/fphys.2025.1578553 (PMC12230438; doi:10.3389/fphys.2025.1578553)
Supplement: Supplementary file 2 [file Table1.pdf]

Supplemental Table S1. Primer pairs used for real-time and *end-point PCR.

Gene Symbol	Gene	GenBank accession	Sequence of Forward (5'-3') and Reverse (3'-5') primers	Amplicon (bp)
Muc5b	mucin 5, subtype B, tracheobronchial	NM_028801.2	5' ACATCCTGACCAAGAAATGTGC<br>3' GACAAGGGCATCTGCGTAAAG	190
Muc5ac	mucin 5, subtypes A and C, tracheobronchial/gastric	NM_010844.3	5' GTGGTGGAAACTGACATTGG<br>3' CATCAAAGTTCCCACACAGG	115
Cre	Cre recombinase	-	5' TGCCTGCATTACCGGTCG<br>3'GCATAACCAGTGAAACAGCATTGCTG	321
**Bdnf*	*brain-derived neurotrophic factor Forward2*	NM_001285422.1	5' GATTGTGTTTCTGGTGAC<br>3' TCAGGTCATGGATATGTCCAA	1400
Actb	actin, beta	NM_007393.5	5' CTGTGGCATCCATGAAACTACA<br>3' GTAATCTCCTTCTGCATCCTGTCA	141
RPL13A	*ribosomal protein L13a*	NM_012423.4	5' GGCCCCTACCACTTCCG<br>3' ACTGCCTGGTACTTCCA	251

BDNF	brain derived neurotrophic factor	NM_170735.6	5’ CTCGTGACAGCATGAGCAGAG<br>3’ GACATGCAGTGTTTCCCCCAA	157
MUC5AC	mucin 5AC, oligomeric mucus/gel-forming	NM_001304359.2	5’ CGAGATCATCTTCAACAACAAGG<br>3’ GTTGGCAAACTTGCTGAAGG	168
IL13RA1	interleukin 13 receptor subunit alpha 1	NM_001560.3	5’ AGGAATACCAGTCCCGACAC<br>3’ TGGAATCCTTCACTTTGGTC	138
IL13RA2	interleukin 13 receptor subunit alpha 2	NM_000640.3	5’ TCTTGGAAACCTGGCATAGG<br>3’ TGCCTCCAAATAGGGAAATC	146
IL4R	interleukin 4 receptor	NM_000418.4	5’ AACGACCCGGCAGATTTCAG<br>3’ AGGAGTTGTGCCACTTGGTG	174
